# Osteopontin activity modulates sex‐specific calcification in engineered valve tissue mimics

**DOI:** 10.1002/btm2.10358

**Published:** 2022-06-15

**Authors:** Megan E. Schroeder, Dilara Batan, Andrea Gonzalez Rodriguez, Kelly F. Speckl, Douglas K. Peters, Bruce E. Kirkpatrick, Grace K. Hach, Cierra J. Walker, Joseph C. Grim, Brian A. Aguado, Robert M. Weiss, Kristi S. Anseth

**Affiliations:** ^1^ Department of Chemical and Biological Engineering University of Colorado Boulder Boulder Colorado USA; ^2^ The BioFrontiers Institute University of Colorado Boulder Boulder Colorado USA; ^3^ Department of Biochemistry University of Colorado Boulder Boulder Colorado USA; ^4^ Department of Molecular, Cellular, and Developmental Biology University of Colorado Boulder Boulder Colorado USA; ^5^ Medical Scientist Training Program University of Colorado Anschutz Medical Campus Aurora Colorado USA; ^6^ Materials Science and Engineering Program University of Colorado Boulder Boulder Colorado USA; ^7^ Department of Bioengineering University of California San Diego La Jolla California USA; ^8^ Sanford Consortium for Regenerative Medicine La Jolla California USA; ^9^ Department of Internal Medicine University of Iowa Iowa City Iowa USA

**Keywords:** calcification, osteopontin, sex differences, synthetic hydrogels, valvular interstitial cells

## Abstract

Patients with aortic valve stenosis (AVS) have sexually dimorphic phenotypes in their valve tissue, where male valvular tissue adopts a calcified phenotype and female tissue becomes more fibrotic. The molecular mechanisms that regulate sex‐specific calcification in valvular tissue remain poorly understood. Here, we explored the role of osteopontin (OPN), a pro‐fibrotic but anti‐calcific bone sialoprotein, in regulating the calcification of female aortic valve tissue. Recognizing that OPN mediates calcification processes, we hypothesized that aortic valvular interstitial cells (VICs) in female tissue have reduced expression of osteogenic markers in the presence of elevated OPN relative to male VICs. Human female valve leaflets displayed reduced and smaller microcalcifications, but increased OPN expression relative to male leaflets. To understand how OPN expression contributes to observed sex dimorphisms in valve tissue, we employed enzymatically degradable hydrogels as a 3D cell culture platform to recapitulate male or female VIC interactions with the extracellular matrix. Using this system, we recapitulated sex differences observed in human tissue, specifically demonstrating that female VICs exposed to calcifying medium have smaller mineral deposits within the hydrogel relative to male VICs. We identified a change in OPN dynamics in female VICs in the presence of calcification stimuli, where OPN deposition localized from the extracellular matrix to perinuclear regions. Additionally, exogenously delivered endothelin‐1 to encapsulated VICs increased OPN gene expression in male cells, which resulted in reduced calcification. Collectively, our results suggest that increased OPN in female valve tissue may play a sex‐specific role in mitigating mineralization during AVS progression.

## 1 INTRODUCTION

Aortic valve stenosis (AVS) is a cell‐mediated progressive disease that affects 2.8% of the US population, but males have a twofold excess risk of developing this degenerative disease.^1^, ^2^, ^3^ Macroscopic and microscopic analyses of stenotic human aortic valves demonstrate that females exhibit more fibrosis whereas males show a higher propensity for calcification during the progression of aortic valve disease.^4^, ^5^, ^6^ Likewise, recent clinical data further support that females have increased hemodynamic dysfunction that results in more severe AVS, yet have lower calcification relative to males.^7^ The underlying biological factors that determine sex differences in cardiac disease and more specifically, aortic valve stenosis remain poorly understood and are multivariate.^8^, ^9^ Identifying the molecular and cellular mechanisms involved in valve fibrosis and calcification is key to understanding AVS progression and developing future precision treatments that consider patient sex.

Valvular interstitial cells (VICs) are known to be the primary mediators of calcification in the aortic valve, and the sex of VICs may impact how calcification manifests and evolves in male and female valve tissue.^10^ In vitro results suggest VICs activate to a pro‐calcific osteoblast‐like phenotype in response to osteogenic cues, such as matrix stiffening, serum phosphate levels, and pro‐calcific cytokines.^11^, ^12^ However, sex is a variable that influences VIC phenotype. For example, sex‐specific in vitro models of valve disease have been used to explore sex differences in early osteogenic markers, gene expression, inflammatory cues in patient sera, and the role of X‐chromosome inactivation in VIC phenotype response.^13^, ^14^, ^15^, ^16^ Additionally, significant decreases in gene expression have been observed in vitro for pathways involved in calcification and ossification in female VICs relative to males.^14^ While it is debated if VICs become true osteoblasts, VICs express markers of osteogenesis including the transcription factor RUNX2, an early marker, as well as osteocalcin (OCN), and osteopontin (OPN).^17^ Although there is evidence that female VICs in their local microenvironment are inherently less predisposed to calcification, the processes behind these observed dimorphisms remain unclear.

OPN is a bone sialoprotein that is widely used as a marker of the osteogenic phenotype, but it is also considered to have anti‐calcific properties.^18^ Prior work has shown OPN likely mediates calcification of the aortic valve microenvironment, as OPN is present to varying degrees in calcified valves, but absent or very low in healthy valve tissue.^19^ Recent clinical data suggest that OPN plasma levels may be a biomarker for severity of chronic heart failure, as high levels correlate with increased risk of mortality^20^ and severity of aortic valve calcification,^21^ yet may be cardioprotective post‐MI.^22^ Studies demonstrate OPN as a potential inhibitor of calcification, as OPN is an acidic phosphoprotein that binds calcium to regulate apatite crystal growth.^23^, ^24^, ^25^ OPN has several post‐translational modifications, including cleavage and phosphorylation sites, lending OPN to bind calcium with high affinity and co‐localize to regions of biomineralization extracellularly.^26^ OPN also plays a role in other calcific diseases and can modulate the response of multiple cell types^27^, ^28^ to prevent, or reverse, ectopic calcification, depending on its phosphorylation state.^29^, ^30^ As such, the pleiotropic role of OPN in regulating calcification has yet to be fully characterized in different contexts, with limited understanding as to how OPN regulates sexually dimorphic disease progression in valve tissue. Previous studies have suggested that OPN expression is enhanced by endothelin‐1 (ET‐1) in cell types such as cardiomyocytes and osteoblasts,^31^, ^32^ yet other work suggests ET‐1 inhibition decreases OPN mRNA expression but also reduces calcification.^33^ Thus, while the role of ET‐1 and OPN in valve calcification remains unknown, ET‐1 offers an upstream target to control OPN and investigate its effects on the distinct pro‐fibrotic versus pro‐calcific AVS phenotypes.

For mechanistic studies involving VICs, hydrogel culture systems have become valuable tools to selectively activate VICs and investigate their transition to myofibroblasts and osteoblast‐like phenotypes.^15^, ^34^, ^35^ For example, the VIC osteoblast‐like phenotype, as indicated by expression of RUNX2, can be promoted when cultured within 3D gelatin methacrylate and hyaluronic acid hydrogel when exposed to osteogenic environmental cues.^36^ Alternatively, VICs cultured on compliant substrates more readily activate to an osteoblast‐like phenotype, as measured via OCN‐positive nodule formation.^37^ Furthermore, soft 1‐kPa 3D hydrogels promoted the osteoblast‐like phenotype of VICs relative to a stiffer 3‐kPa hydrogel formulation.^38^ Taken together, VIC osteogenesis may be investigated in vitro using soft 3D materials and may be used to clarify the effects of OPN on VIC phenotype.

Here, we aimed to explore the sex‐specific role of OPN in mediating calcification of aortic valve tissue. We hypothesize that OPN reduces calcification in female valve leaflets relative to male valves. To this end, we use a degradable PEG‐based hydrogel with an interpenetrating network (IPN) of collagen type I that mimics aspects of the native valve microenvironment. We have shown previously that incorporating collagen type I, a component of valve tissue, into these PEG hydrogels allows for cell‐mediated mineralization processes to occur in this synthetic system.^39^ We employ this 3D hydrogel culture system as a precision biomaterial^40^ to explore the sex‐specific role of OPN in regulating sexually dimorphic calcification. We initially characterized human diseased aortic valve tissue to confirm trends observed in clinical data, demonstrating that female aortic valves have reduced calcification and increased OPN. Using our in vitro disease models, we next show female VICs express higher levels of OPN whereas males have a higher degree of calcification. We further observed that OPN localization in female samples shifted from ubiquitous expression throughout the extracellular matrix to localized perinuclear regions that correlated with matrix mineralization. Increased OPN expression and a shift in OPN localization in female VICs treated with calcifying cues suggest that OPN plays a role in reducing calcification of female diseased valve tissue.

## 2 MATERIALS AND METHODS

### 2.1 Frozen tissue section staining

Flash‐frozen human diseased aortic valve tissue sections were purchased from OriGene and stored at −70 °C until use. Patient and available disease information is presented in Table S1. At the time of these studies, three individual female samples and two individual male samples characterized with “aortic valve disease” were available. Five‐micrometer‐thick tissue sections were brought to room temperature and then fixed for 12 min in room temperature 4% paraformaldehyde (Electron Microscopy Sciences) diluted in phosphate‐buffered saline (PBS). Samples were then outlined in PAP pen (Fisher Scientific), hydrated in PBS for 3 × 5 min, and rinsed with DI water for 3 × 2 min in preparation for Von Kossa (VK) staining. Samples were covered in 5% silver nitrate solution and put under UV light for 1 h. Slides were rinsed for 2 min under running DI water and then incubated for 2 min in 5% sodium thiosulfate. Slides were again rinsed for 2 min under running DI water and incubated for 5 min in nuclear fast red solution. A final rinse under running DI water was performed before the sample was dehydrated in 95% alcohol, 2 × 1 min, and then 100% alcohol (2 × 1 min, then 3 min) and cleared in SafeClear (three changes, 2 min). A coverslip was mounted using Permount (Sigma‐Aldrich) and the slide dried for >48 h before imaging.

For immunofluorescence staining, slides were fixed as listed above in 4% PFA and rinsed in PBS 3 × 5 min, before permeabilization with 0.1% TritonX100 (Sigma‐Aldrich) in PBS for 30 min at room temperature. Samples were then blocked in 5% bovine serum albumin (BSA, Sigma‐Aldrich) in PBS for 1 h at room temperature. Primary antibody (1:2000 anti‐COL1A1, ab34710; 1:1000 anti‐RUNX2, ab23981; 1:1000 anti‐OPN, ab8448) was diluted in 5% BSA in PBS and incubated overnight in a humidity chamber at 4°C. Samples were then warmed to room temperature, and rinsed 3 × 5 min with PBST (0.1% Tween20, Sigma‐Aldrich). Secondary antibody was diluted in PBS (1:300 for proteins of interest, 1:1000 DAPI) and incubated at room temperature for 1 h in a humidity chamber. Samples were kept in PBS for unmounted imaging. Porcine valve leaflet tissues were collected within 24 h of slaughter, isolated from the valve cusp, and flash frozen using Tissue‐Tek OCT Compound (VWR) in a cryomold on top of dry ice. Samples were brought to −20°C and sectioned at 30‐μm onto glass slides (Colorfrost plus, Fisher Scientific) and stored at −20°C until use. Samples were prepared for IF staining using the same protocol used for human tissues.

### 2.2 Polymer synthesis

Eight‐arm 40‐kDa poly(ethylene) glycol (PEG) was functionalized with norbornene as described previously.^41^ In brief, amine‐terminated 40‐kDa PEG (JenKem) reacted with 48 molar equivalents of 5‐norbornene‐2‐carboxylic acid (Sigma‐Aldrich), 40 molar equivalents of O‐(7‐Azabenzotriazol‐1‐yl)‐N,N,N,N‐tetramethyluronium hexafluorophosphate (HATU, Chem‐ Impex International, Inc.), and 80 molar equivalents of N,N‐Diisopropylethylamine (DIEA, Sigma‐Aldrich) in N,N‐Dimethylformamide (DMF, Fisher Scientific) overnight. The product was precipitated dropwise into cold diethyl ether (VWR), centrifuged, and washed with diethyl ether. The product was dried by vacuum, resuspended in DI water, dialyzed in 12‐kDa MWCO regenerated cellulose tubing (SpectraPor), and lyophilized. Proton nuclear magnetic resonance imaging of approximately 88% provided confirmation of end group functionalization.

### 2.3 VIC isolation and culture conditions

Female and male porcine hearts (within 24 h of slaughter) were obtained from Hormel Food Corporation. Male and female VICs were pooled, respectively, from 30 individual, sex‐separated hearts. Aortic valve leaflets were extracted and submerged in Earle's Balanced Salt Solution (Sigma‐Aldrich) supplemented with Penicillin/streptomycin (ThermoFisher Scientific) and amphotericin B (ThermoFisher Scientific, 1 μg/ml amphotericin B, 0.82 μg/ml sodium deoxycholate) at 37°C. The tissues were then resuspended in sterilized type II collagenase (Worthington Biochemical Corporation) at 250 U/ml in wash buffer and agitated for 30 min at 37°C. Samples were first vortexed for 30 s and endothelial cells were eliminated by the removal of the supernatant. Samples were digested for a second time in fresh collagenase solution for 70 min and then vortexed for an additional 2 min. A 100‐μm cell strainer was utilized to remove remaining leaflet fragments from the sample and the isolated VICs were resuspended in M199 media (ThermoFisher Scientific) containing 15% fetal bovine serum (FBS, ThermoFisher Scientific), 1.2% penicillin/streptomycin, and amphotericin B (1 μg/mL amphotericin B, 0.82 μg/ml sodium deoxycholate). Isolated VICs were plated on tissue culture polystyrene (TCPS) and expanded for up to 7 days, changing media on alternating days. Cells were frozen in 45% M199 media supplemented with 15% FBS, 50% FBS, and 5% DMSO (Sigma‐Aldrich). All experiments utilized 10% FBS media supplemented with 1.2% penicillin/streptomycin, and amphotericin B (1 μg/ml amphotericin B, 0.82 μg/ml sodium deoxycholate).

### 2.4 VIC encapsulation in PEG + Col

Female or male VICs (P2s) were encapsulated at a concentration of 10 million cells/ml within a matrix metalloproteinase (MMP)‐degradable PEG hydrogel with 1 mg/ml rat tail collagen type I (Fisher Scientific) IPN. PEG + Col hydrogels were prepared from a polymer solution of eight‐arm 40‐kDa PEG norbornene ([enes] = 6 mM), photoinitiator lithium phenyl‐2,4,6‐trimethylbenzoylphosphinate ([LAP] = 1.7 mM), CRGDS adhesive peptide ([thiols] = 1 mM, Bachem), and peptide cross linker KCGPQG↓IWGQCK ([thiols] = 3.5 mM, Bachem) with a thiol:ene ratio of 0.75 in 10X Dulbecco's phosphate‐buffered saline (Thermo Fisher Scientific). Swollen shear storage modulus of the hydrogels was 550 ± 100 Pa for encapsulation studies as measured by oscillatory rheology. When the mixture was prepared, the collagen was added slowly and 200 mM NaOH (Fisher Scientific) was added to obtain a neutral pH. VICs were encapsulated at 10 million cells/ml. Thirty microliters of the polymer solution were pipetted into a 6 × 1‐mm rubber mold on a Teflon sheet, polymerized under 365‐nm 2.5‐mW/cm UV light for 3 min, and then submerged in 10% FBS M199 media overnight prior to experimental treatment.

Osteogenic medium was prepared using 10% FBS M199 supplemented with 10 mM β‐glycerophosphate (Sigma‐Aldrich), 50 μg/ml L‐ascorbic acid (Sigma‐Aldrich), and 1 mM dexamethasone (Sigma‐Aldrich). Calcifying medium was prepared by supplementing OM with 1 mg/ml of calcium chloride (Sigma‐Aldrich). Culture media was refreshed every 3 days.

### 2.5 mRNA isolation and gene expression via RT‐qPCR

To extract mRNA, hydrogels were digested in type II collagenase (Worthington Biochemical Corporation) at 2 mg/ml. Samples were centrifuged for 5 min at 1000 rpm and supernatant was removed. The sample was resuspended in 10% FBS phenol‐free M199 media (Thermo Fisher Scientific) and strained through a 100‐μm cell strainer. Samples were centrifuged again at 1000 rpm for 5 min. Supernatant was removed and the remaining pellet was lysed for mRNA isolation. mRNA isolation was accomplished using an RNAeasy Micro Kit (Qiagen) per the manufacturer's instructions. An ND‐1000 Nanodrop Spectrophotometer evaluated the quality and concentration of the mRNA. cDNA was synthesized with an Eppendorf Mastercycler from a mixture of RNAse‐free water, RNA, and iScript Reverse Transcription Supermix (Bio‐Rad). Relative mRNA levels were measured using SYBR Green reagents (Bio‐Rad) with an iCycler machine (Bio‐Rad) and the housekeeping gene L30 was used for normalization (Table 1).

**TABLE 1:** Primer sequences for RT‐qPCR

Gene	Forward primer (5′‐3′)	Reverse primer (5′‐3′)
L30	AGATTTCCTCAAGGCTGGGC	GCTGGGGTACAAGCAGACTC
RUNX2	AACAACCACAGAACCACAAG	TGACCTGCGGAGATTAACC
OPN	GCGTCTTCTGAGATCAACTG	CACTATACATTCACCAACTAAGC

### 2.6 Western blots for protein detection

Hydrogels were digested as described for mRNA isolation and the cell pellet was resuspended in RIPA buffer (ThermoFisher Scientific) supplemented with Protease/Phosphatase inhibitor and ETDA (ThermoFisher Scientific) at a 1:100 concentration to collect protein lysate. Protein concentrations were determined with microBCA Kit (BioRad) in order to load the same protein content per lane. Lysates were subsequently combined with 5X Laemmli sample buffer and assessed through SDS‐PAGE gel electrophoresis and subsequent western blot analysis. Protein electrophoresis was run using 4%–20% Mini‐PROTEAN TGXTM precast gels (Bio‐Rad) and protein was transferred onto polyvinylidene difluoride membranes (Bio‐Rad). The membrane was rinsed and covered in 5% milk in TBST with diluted primary antibody (1:1000 anti‐OPN ab8448, 1:10,000 anti‐histone 3 [HIS3] ab1791) incubating overnight at 4°C. Membranes were rinsed 3 × 10 min in 5% milk in TBST, incubated with secondary conjugated antibody (Jackson ImmunoResearch; 1:5000 anti‐Rb for OPN and 1:10,000 for HIS3) diluted in 5% Milk in TBST and rocked at room temperature for 1 h. Chemiluminescence was detected using Pierce ECL Plus solution (ThermoFisher Scientific) and ImageQuant LAS 4000 detector was used to assess relative protein expression to HIS3.

### 2.7 Embedding and sectioning of hydrogel samples

Hydrogels were fixed in 10% formalin (Sigma‐Aldrich) for 30 min, rinsed with multiple changes of PBS, and further rinsed in Tissue‐Tek OCT Compound (VWR). The samples were then submerged in OCT for 48 h at 4°C. Samples were centered in a cryosectioning mold filled with OCT and were frozen in 2‐methylbutane (Sigma‐Aldrich) cooled by dry ice. Samples were stored at −70°C and brought to −20°C at time of use. Samples were sectioned (30 μm for hydrogels) using a Cryostat (CM1850, Leica) and placed on glass slides (Colorfrost plus, Fisher Scientific) and stored at −70°C.

### 2.8 Calcium assay

Calcium content of samples was quantified using a Quantichrome Calcium Assay Kit (BioAssay Systems) following the manufacturer's instructions. For sample preparation, cell‐laden gels are rinsed with PBS (no calcium added) for 3 × 5 min, flash frozen, and lyophilized until dry. Then, 100 μl of 1 M HCl is added to the dried samples and stored at 4°C for 48 h. Samples were diluted with 1 M HCl to be within the working range of the assay, and 5 μl of samples and standards were pipetted into a 96‐well plate to compare sample concentration to a standard curve. Two‐hundred microliters of working reagent were added to each well and incubated for 3 min at room temperature. Absorbance was measured at 612 nm and the standard curve was used to determine the concentration of calcium in each sample.

### 2.9 Image acquisition and analysis

Immunofluorescence (IF) images were acquired using a 20x air objective on a Zeiss LSM 710 confocal microscope. At minimum, 6‐μm z‐stacks were collected using four z‐steps. IF images were analyzed with FIJI^42^ by creating an ROI and measuring the mean fluorescence intensity of each image.

For cell:ECM intensity ratios, a custom MATLAB (2017a) script was applied to analyze signal localization. Briefly, a median intensity z‐projection image was created to improve nuclear segmentation and to accurately represent the OPN signal throughout the entire image volume. Nuclei were identified using DAPI signal, and those regions were dilated to include a small area of pixels surrounding each nucleus (“cellular” signal). Mean OPN signal intensities were then calculated for two distinct pixel subsets in each image: “cellular” pixels (identified by DAPI), and “ECM” pixels (all non‐cellular pixels in the image). The data are presented as a ratio of mean OPN intensity values, which were calculated by dividing the cellular mean intensity by the ECM mean intensity (i.e., cell:ECM).

Histological samples stained with VK were imaged at ×20 with a Nikon Eclipse TE300 microscope and color camera. VK histology images were quantified using FIJI to measure integrated image intensity.^30^ Briefly, images were converted from RGB to 8‐bit grayscale, then a threshold was uniformly applied across conditions to yield binary images. Image intensity was quantified using the measure function and used for statistical analysis.

Additionally, these binary images were processed using a radially averaged autocorrelation function in FIJI to calculate the correlation length of VK puncta in each image (Figure S1). Correlation length is determined by integrating the radial autocorrelation function, and describes the maximum radial distance at which features (i.e., VK puncta vs. surrounding tissue) are well segregated.^43^, ^44^ In other words, the correlation length is a measure of feature size; images with larger features will correlate at greater distances. Pixel values were converted using a measured conversion of 2.73 pixels/μm. Additional discussion regarding the utility of spatial autocorrelation measurements is included in the Supporting Information Material S1.

### 2.10 Rheological measurements

Swollen hydrogel mechanical properties were assessed on a DHR3 rheometer (TA Instruments) using frequency sweep and strain sweeps. This ensured that measurements fell within the linear viscoelastic regime. Studies were done at 37°C with an 8‐mm standard geometry Peltier Plate tool coated with adhesive 600/P1200 sandpaper to prevent slippage. PEG and PEG + Col hydrogel formulations were not statistically different in measured shear storage modulus.

### 2.11 Statistical analysis

Data are presented as mean ± standard deviation. A minimum of three technical replicates were used throughout the study unless otherwise stated in appropriate figure captions. Data were analyzed using Graph pad (Prism 8), either by unpaired *t* test, with a Welch's correction when appropriate, or ordinary one‐way analysis of variance (ANOVA) was used with a Dunnett's multiple comparison test. Two‐way ANOVA was used with either a Dunnett's multiple comparison test, or Tukey's multiple comparison test. Statistical tests and post hoc analyses are indicated in the appropriate figure captions.

## 3 RESULTS

### 3.1 Human valve tissue from patients with aortic valve disease shows potential sex differences in size and distribution of calcification

**FIGURE 1:**
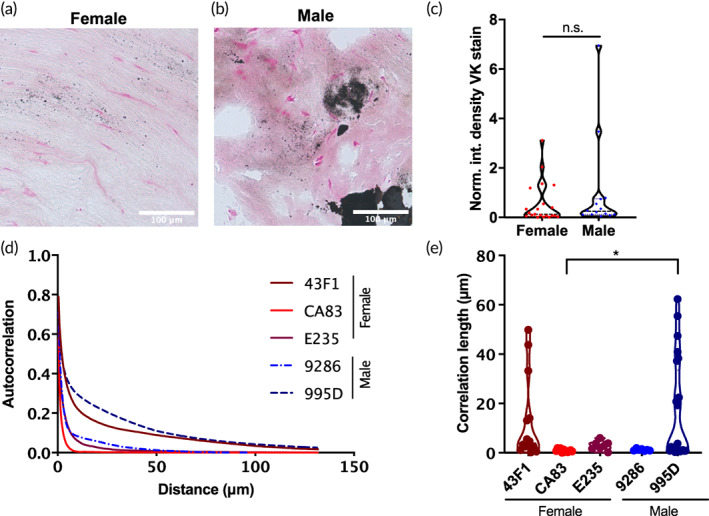
Human valve tissue from patients with aortic valve disease shows potential sex differences in size and distribution of calcification. **(**a) A representative image of a histological section from a female diseased valve stained with Von Kossa (VK) shows small, positive punctae of VK staining. (b) A representative image of a male histological section of diseased valve shows large regions of positive VK staining. (c) Normalized integrated density of total positive VK staining between female and male tissue sections. (d) Radial autocorrelation profiles of positive VK staining in human diseased valve tissue, and (e) correlation length analysis per patient sample. Female data are presented in red, males in blue. For patient‐specific details, refer to Supporting Information Material S1 (*n* = 3 females, 2 males: seven images per patient sample minimum; *p < 0.05 as determined via one‐way ANOVA; scale bar: 100 μm).

Clinical data suggest that female patients have reduced severity of calcification in AVS, so we first sought to identify these sexual dimorphisms in patient tissue samples via histological staining of calcification. Using flash‐frozen aortic valve tissue sections from male and female patients diagnosed with aortic valve disease available at the time of this study (Table S1), we stained valve sections with VK to assess phosphate deposition and saw that female patients had reduced overall calcification within the available data set. Representative images of histological sections stained with VK show small, positive black punctae in female tissue relative to the larger black nodules observed in male tissue (Figure 1a,b). While the overall normalized and integrated density of the VK staining was not significantly different between female and male patients, there was a trend of increased positive staining in male patient samples (Figure 1c).

To better assess the distribution and spatial organization of VK‐positive punctae, we utilized radial autocorrelation analysis to determine the correlation length of each sample. Radial autocorrelation analysis calculates the average “similarity” between pixels compared across increasing radial distances; two pixels of the same binary value have an autocorrelation of 1, and pixels of opposite binary values have an autocorrelation of −1. Generally, autocorrelation curves begin near 1 (as even images with small feature size will autocorrelate highly at short comparison distances) and decrease quickly with increasing spatial lag. Samples with large features (i.e., increased frequency and density of VK‐positive signal) autocorrelate highly over longer distances, which can be summarized by integrating the area under these curves to determine the correlation length (see Figure S1 for more details). Autocorrelation curves were generated for female and male tissue sections (Figure 1d), and clearly illustrate differences in spatial organization of calcification between heavily calcified samples compared to sparser VK staining. Integrating these curves displays these same trends (Figure 1e), with measured correlation lengths >10 μm only present in heavily calcified samples. Notably, male patient 9286 suffered from aortic insufficiency originating from aortic root dilation and did not have stenotic disease, potentially explaining the minimal calcification in this sample. Overall, size distribution and organization of calcification in female valves trends toward smaller, sparser aggregates compared to male tissues. However, some female samples do demonstrate substantial regions of dense calcification. These data suggest that although diseased female valve tissue may be equally calcified as corresponding male valve tissue (as there was no overall significant difference in the total integrated density of positive VK staining), the mechanisms of calcium deposition may be sexually dimorphic (e.g., suppressing the growth of large mineral deposits or promoting a higher nucleation rate in females).

### 3.2 Female human diseased valve tissue sections have increased COL1A and OPN staining relative to male tissue sections

**FIGURE 2:**
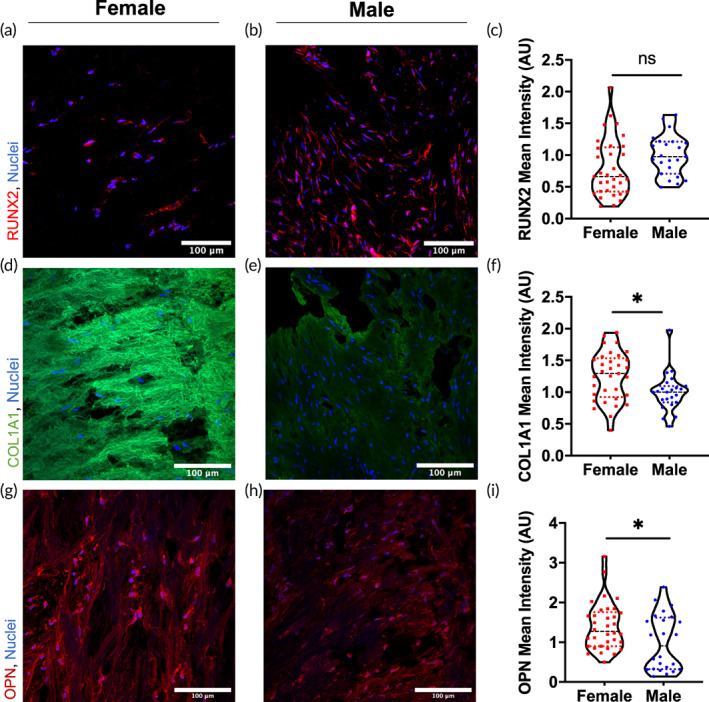
Female human diseased valve tissue sections have increased COL1A and OPN staining relative to male tissue sections. A representative image of RUNX2 (red) and nuclei (blue) staining in female (a) and male (b) diseased valve tissue. (c) Quantification of RUNX2 mean fluorescence intensity indicates no significant difference in intensity between female and male samples. (d) Representative image of a female diseased valve tissue section stained for COL1A1 (green) and nuclei (blue), relative to male tissue (e). (f) Quantification of COL1A1 mean fluorescence intensity indicated a significant increase in staining in female valve tissue relative to male. (g) Representative image of female diseased valve section stained for OPN (red) and nuclei (blue) relative to male tissue (h). (i) Quantification of the OPN mean fluorescence intensity shows increased OPN staining in the female valve tissue sections compared to male (*n* = 3 females, 2 males; 10 images per patient sample minimum. *p < 0.05 as determined via unpaired *t* test; scale bar: 100 μm).

To explore the dimorphisms observed in calcification between female and male valve tissue, we next stained aortic valve tissue from patients with aortic valve disease for other markers of calcification. RUNX2 was used as an early marker of the osteogenic phenotype, to further characterize differences in pro‐calcific markers within diseased valve tissue. Female tissue sections stained for RUNX2 (red) and nuclei (blue) demonstrated decreased staining (Figure 2a) with respect to male tissue (Figure 2b), albeit this decrease was not significantly different (Figure 2c).

We then stained for levels of collagen type I (COL1A1), an extracellular matrix molecule that plays a key role in valve stiffening, the VIC fibrotic phenotype, and calcification. Female tissue sections stained for COL1A1 (green) and nuclei (blue) (Figure 2d) showed intense staining for COL1A1 revealing a disorganized and fibrous collagen matrix, which contrasted with the more diffuse and lower levels observed in the male tissue sections (Figure 2e). Quantification of the mean fluorescence intensity for COL1A1 staining showed a significant ~25% increase in COL1A1 signal in female tissue sections relative to male tissue sections (Figure 2f).

While OPN is an osteogenic marker, it may also be involved with inhibiting calcification growth.^26^ We next stained human tissue sections for OPN and noted that female tissue sections had increased OPN staining throughout the valve (Figure 2g) when compared to male tissue sections (Figure 2h). Quantification of the mean OPN fluorescence intensity between female and male tissue sections revealed a significant 1.4‐fold increase in OPN protein staining in female samples relative to male (Figure 2i). These results suggest that VICs in female valve tissue may be secreting more OPN or the matrix microenvironment may be sequestering more of the secreted OPN compared to male valve tissue, as collagen is a known binder of OPN.^45^ Staining for OPN was repeated using freshly isolated, flash‐frozen porcine aortic valves (Figure S2). No significant differences between sexes were observed in OPN levels of healthy porcine tissues. Combined, these results corroborate clinical observations of sexual dimorphisms in human aortic valves by echocardiographic characteristics^7^ and that sex differences in disease are observable at the tissue level.

### 3.3 Female VICs encapsulated within PEG + Col hydrogels have increased OPN gene expression

**FIGURE 3:**
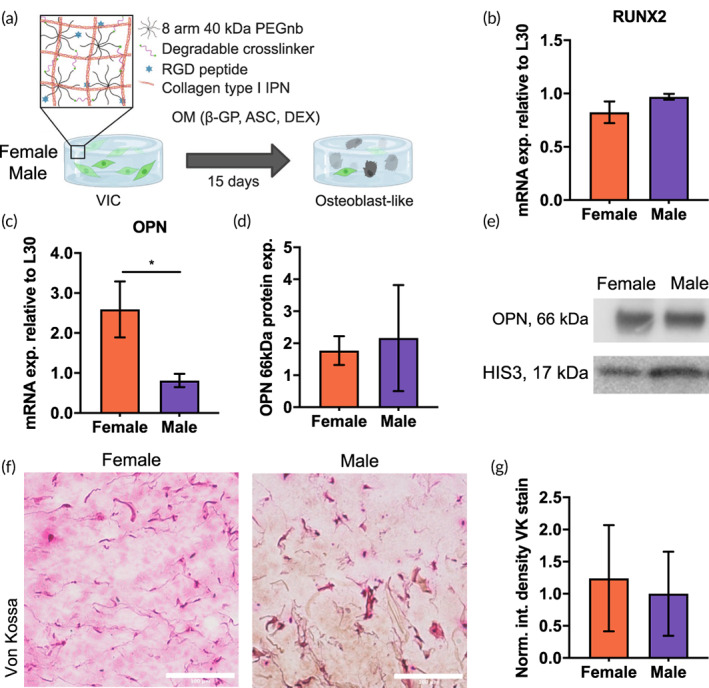
VICs encapsulated within 3D PEG‐based hydrogels preserve increased OPN observed in female human tissue. (a) Experimental schematic shows sex‐separated VICs encapsulated within degradable hydrogels synthesized from PEG + collagen type I (Col) IPN and exposed to osteogenic medium (OM; 10 mM βGP, 50 mg/ml ASC, and 1 mM DEX) for 15 days. (b) RUNX2 mRNA expression of female and male VICs, relative to L30 control. (c) OPN mRNA expression increased in female VICs relative to males, relative to L30 control (*n* = 3 biological and technical replicates for qPCR analysis). (d) Full‐length OPN protein (66 kDa) expression from encapsulated VICs in female and male samples (*n* = 3 biological replicates run in same blot). (e) Representative western blot lanes for OPN (66 kDa) and normalization protein (HIS3, 17 kDa) for female and male samples. (f) Representative image of a histological section from female (left) sample stained with Von Kossa (VK), relative to stained male section (right), which displayed no discrete positive staining punctae in either sex. (g) Quantification of normalized total integrated density of positive VK staining between female and male sectioned samples shows no significant differences (*n* = 3 individual hydrogels of each sex per assessment, *p < 0.05 as determined via unpaired *t* test; scale bar: 100 μm).

We next encapsulated porcine VICs within 3D degradable hydrogels that recapitulate key aspects of the aortic valve microenvironment, including relevant osteogenic and calcific phenotypes depending on media conditions.^39^ Our goal was to test if sex differences were observed in our in vitro model compared to the aortic valve tissue samples and then investigate any role of OPN in inhibiting matrix calcification. To this end, we selected a poly(ethylene glycol)(PEG)‐norbornene hydrogel (G′ ≈ 500 Pa) crosslinked with an MMP degradable cross‐link (KCGPQG↓IWGQCK) and fibronectin‐derived CRGD ligands to promote VIC integrin binding to the network. Additionally, we incorporated collagen type I to create an interpenetrating proteinaceous network within the largely PEG‐based hydrogel. Collagen type I is a native extracellular matrix component present throughout the valve and is known to play a role in matrix calcification. Sex‐separated VICs were encapsulated within the PEG + Col hydrogel system and exposed to osteogenic medium (OM; 10 mM βGP, 50 μg/ml ASC, and 1 mM DEX) for 15 days (Figure 3a). Samples were then collected and processed to measure any sex‐specific differences in osteogenic markers and calcification. Female VICs expressed lower levels of RUNX2 mRNA, but this was not significantly different from the male cells (Figure 3b).

We next measured relative OPN mRNA levels between the female and male VICs at Day 15 in OM and observed that female VICs expressed 3.2‐fold more OPN mRNA compared to males (Figure 3c). Given this difference at the gene level, we then measured protein levels of OPN in female and male samples using western blots. These data showed no significant data between sexes at this timepoint (Figure 3d,e).

We then sectioned the VIC‐laden hydrogels and stained them with Von Kossa to measure any differences in phosphate deposition between sex‐separated VICs after 15 days in OM. The VK stained sections showed no discernable differences between sexes (Figure 3f) as neither condition resulted in positive black staining deposition. These staining results were quantified by integrated density analysis. While female samples had a lower overall positive VK staining, there were not significant differences in staining based on sex (Figure 3g). While the degradable PEG + Col hydrogel system resulted in elevated OPN gene expression in female VICs, matrix calcification was not observed at this time point under these culture conditions in osteogenic medium alone.

### 3.4 VICs cultured in PEG + Col IPN hydrogels in the presence of calcifying medium recapitulate the sexual dimorphisms observed in valve tissue calcification

**FIGURE 4:**
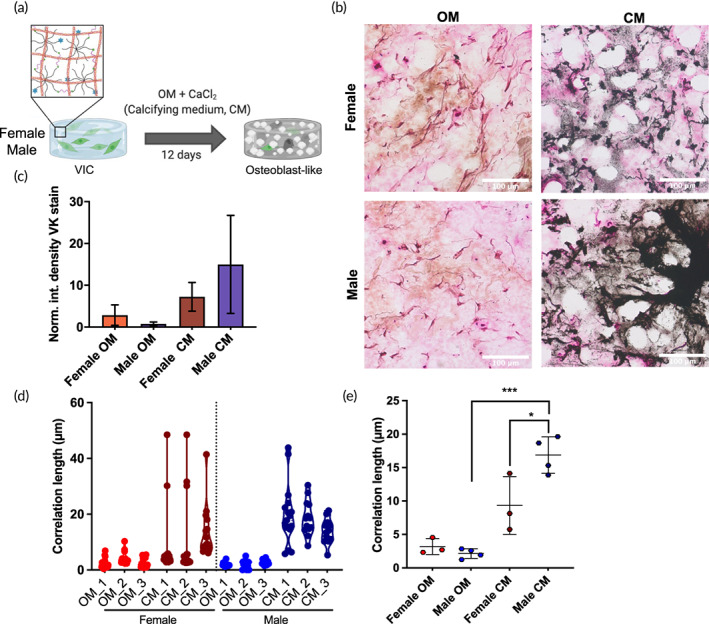
PEG + Col hydrogels in calcifying medium show reduced calcification in female samples. (a) Experimental schematic shows female or male porcine VICs encapsulated within degradable hydrogels synthesized from PEG + Col and exposed to calcifying medium (CM, OM [osteogenic medium] + 1 mg/ml CaCl_2_) for 12 days. (b) Representative images of histological sections stained with Von Kossa (VK) for female and male VICs within the PEG + Col hydrogel in CM relative to OM. Females (top) displayed small punctae of positive VK staining with CM, where males (bottom) showed large areas of positive punctae in samples treated with CM. (c) Integrated density of VK staining for female and male encapsulated VICs in OM or CM after 12 days in culture. (d) Correlation length analysis of VK staining in individual female and male samples treated for 12 days in OM, or CM. (e) Sex‐averaged correlation lengths (*p < 0.05, ***p < 0.001 as determined via one‐way ANOVA, scale bar = 100 μm)

We next cultured VICs within a 3D in vitro hydrogel system while exogenously delivering media components that promotes mineralization over an accelerated time scale (<2 weeks) to observe any sexual dimorphisms in matrix calcification.^39^ Female or male porcine VICs were encapsulated within PEG + Col IPN hydrogels and cultured in calcifying medium (CM, OM + 1 mg/ml CaCl_2_) for 12 days, prior to assessing any changes in matrix mineralization (Figure 4a). Calcium deposition was quantified via a colorimetric assay at Days 7 and 12 in OM or CM. Calcium deposition was not significantly different between sexes; however, treatment with CM resulted in a significant increase in detected calcium between Days 7 and 12 for both sexes relative to samples cultured in OM (Figure S3).

We next assessed phosphate deposition via VK staining to identify other differences between matrix mineralization with sex (Figure 4b). Encapsulated female samples demonstrated little increase in overall black staining, but small black punctae in CM conditions were observed (top). Male samples displayed an increase in large black VK staining of samples in CM relative to OM (bottom). Total VK staining between female and male samples was quantified by integrated density analysis (Figure 4c). No significant sex differences were observed between total VK staining in VICs cultured in OM, but there was ~3x more VK staining in males cultured in CM relative to females in CM.

The same radial autocorrelation analysis for VK punctae that was utilized for human tissues (Figure 1) was used to calculate the correlation lengths for VK staining in female and male VICs cultured in PEG + Col gels in CM compared to OM (Figure 4d). These data are in good agreement with the human tissue analysis and show high repeatability across replicates, demonstrating the utility of this strategy for recapitulating disease‐related sexual dimorphisms. As with the human samples, heavily calcified samples (CM conditions) have correlation lengths >10 μm. Strikingly, sex‐specific differences are detectable with this analysis (Figure 4e), as male VICs in CM gels have correlation lengths that are approximately 1.5‐fold greater than their female counterparts (16 vs. 9 μm). These results suggest that in vitro cultures can mimic aspects of sexually dimorphic calcification observed in humans with AVS as represented by the extent and spatial distribution of VK staining.

### 3.5 OPN localization shifts in female VICs cultured in PEG + Col hydrogels and treated with calcifying medium

**FIGURE 5:**
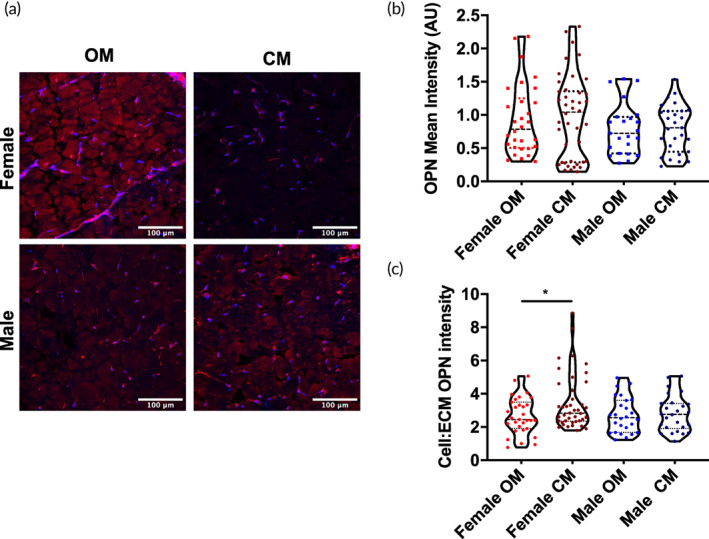
OPN distribution shifts from the ECM to nuclear regions in encapsulated female VICs cultured in calcifying medium. (a) Representative histological sections with immunofluorescent (IF) staining for OPN (red) and nuclei (blue) for female (top) and male (bottom) conditions, in OM (osteogenic medium) or CM (calcifying medium). (b) Quantification of OPN mean fluorescence intensity indicates no significant differences in OPN intensity in calcified samples of either sex. (c) OPN fluorescence intensity was measured as a function of nuclear distance. Results indicate a significant increase in the cell:ECM ratio for OPN signal in female samples treated with CM (*p < 0.05 as determined with one‐way ANOVA, scale bar: 100 μm).

Female or male VICs were encapsulated within PEG + Col IPN hydrogels and exposed to CM (OM + 1 mg/ml CaCl_2_) for 12 days. Sectioned samples were stained for OPN (red) and cell nuclei (blue) (Figure 5a). Female VICs (top) demonstrated a high intensity of OPN staining in the OM conditions localized within the extracellular matrix (ECM) of the hydrogel section, rather than around the nuclei. In contrast, for female VICs in the CM condition, OPN was less intense in the ECM, but localized around the nuclei of the cells. Male VIC samples (bottom) demonstrated little to no change in OPN intensity or localization between OM and CM conditions at Day 12.

Quantification of overall OPN fluorescence intensity indicated there was a higher signal in female VICs cultured in CM, although this was not significantly increased relative to all other conditions (Figure 5b). We then quantified the intensity of OPN signal with respect to distance from the nuclei using a custom MatLab script (Figures 5c and S4) for the OM or CM conditions. Mapping the cell:ECM mean intensity ratio of signal showed that while there was no significant differences between males in OM and CM, the change in OPN localization between females cultured in OM and CM was significantly different. This indicated that local changes in the OPN distribution may play a significant role in matrix mineralization in valve tissue; the intensity of the OPN signal near the nuclei for female VICs was significantly increased in CM relative to OM.

### 3.6 Exogenous treatment of ET‐1 results in sex‐specific increase in OPN expression while reducing matrix calcification

**FIGURE 6:**
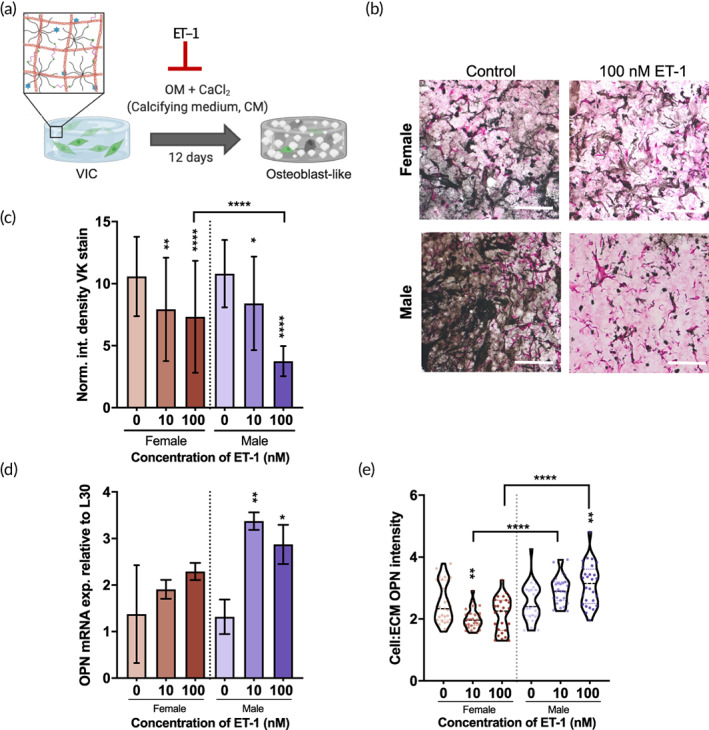
ET‐1 treatment reduces calcification while increasing OPN gene expression. (a) Experimental schematic shows female or male porcine VICs encapsulated within degradable hydrogels synthesized from PEG + Col and exposed to calcifying medium (CM, OM [osteogenic medium] + 1 mg/ml CaCl_2_) supplemented with exogenous ET‐1. (b) Representative images of histological sections stained with Von Kossa (VK) for control (CM only) versus ET‐1 (CM + 100 nM ET‐1) treated female or male samples after 12 days in culture. (c) Quantification of VK staining for female or male histological sections of samples cultured for 12 days in CM + indicated concentration of ET‐1 (*n* = 2 biological replicates minimum, >14 FOV per replicate). (d) OPN gene expression of female or male samples in CM + ET‐1 for 3 days, relative to L30 control (*n* = 3 biological and technical replicates). (e) Quantification of OPN cell:ECM ratio for female or male histological sections stained for OPN after 12 days in culture in CM + indicated concentration of ET‐1 (*n* = 3 biological replicates, >5 images per replicate) (*p < 0.05, **p < 0.01, ***p < 0.001, ****p < 0.0001; scale bar = 100 μm).

To test our hypothesis that OPN was mediating the calcification within the 3D hydrogel system, we sought to induce OPN gene expression in VICs via exogenous stimulation with ET‐1. ET‐1 has been shown to induce OPN expression in cell types such as cardiomyocytes^46^ and osteoblasts.^47^ Female or male VICs were encapsulated within the PEG + Col hydrogel system and cultured for 12 days in the presence of calcifying stimuli (CM) with either 0, 10 nM, or 100 nM ET‐1 (Figure 6a). Hydrogels sectioned and stained with VK at the 12‐day mark showed reduced calcification with ET‐1 treatment (Figure 6b). Quantification of these results demonstrates a decrease in VK integrated density signal with increasing concentration of ET‐1 in the presence of CM (Figure 6c). Interestingly, there was a significant difference between female and male VICs at the 100 nM ET‐1 condition. Male VICs in CM treated with 100 nM ET‐1 resulted in a 1.9‐fold decrease in VK staining relative to female under the same treatment conditions.

To determine if this reduction in calcification was due to increased OPN expression in response to ET‐1 treatment, we assessed OPN levels of VICs within the hydrogels. Due to the highly mineralized nature of the samples at 12 days, isolating quality mRNA was difficult; therefore, we used an earlier 3‐day timepoint to assess OPN gene expression levels. Female VICs had non‐significant differences in OPN gene expression due to ET‐1 treatment; however, male VICs demonstrated an increase in OPN expression at 10 and 100 nM ET‐1, relative to the control condition (Figure 6d). Interestingly, this trend was not observed in female and male VICs cultured on TCPS (Figure S5A), indicating a potential role of dimensionality or substrate stiffness in OPN expression.

To assess the level of OPN at the later 12‐day timepoint, the cell:ECM fluorescence intensity was measured from sectioned and stained samples (Figure 6e). Female sample treated with ET‐1 did not result in a measurable change in OPN localization, relative to the control, whereas male samples treated with ET‐1 at both the 10 and 100 nM condition resulted in a shift of OPN localization from regions of the ECM to perinuclear regions. However, there was no significant difference in OPN signal intensity overall between sex (Figure S4B).

## 4 DISCUSSION

Clinical results and results presented herein from the analysis of human diseased valve tissues guided the development of an in vitro hydrogel model that recapitulates several key sexual dimorphic responses of VICs. Our methods use a soft, MMP‐degradable PEG‐based hydrogel that recapitulates some key clinically observed sex differences in human valves using sex‐separated porcine VICs to test hypotheses in vitro. We asked whether OPN, a bone sialoprotein associated with calcification inhibition, plays a sex‐specific role in mediating valve microenvironment calcification. Our results suggest that calcification in female diseased valve tissue tends to involve smaller, micro‐calcific deposits, especially relative to larger nodules observed in male valve tissue. Additionally, we noted a shift in localization of OPN from the ECM to the nuclear region in encapsulated female VIC samples, which may contribute to reduced calcification observed in both female valve tissue and encapsulated female VICs. Further, ET‐1 delivered exogenously increased OPN mRNA gene expression in encapsulated male VICs, resulting in reduced calcification.

Our data show that while female diseased valves have lower bulk calcification, there is a significant amount of microscale calcification, especially compared to the larger mineral depositions found in male valve tissue (Figure 1). Clinical data from female and male patients with the same degree of AVS severity show reduced computed tomography aortic valve calcium score (CT‐AVC) for female patients,^7^, ^47^ indicating reduced calcification in female aortic valve leaflets relative to male patients. Modern clinical imaging techniques, such as CT or ultrasound, do not have the size scale resolution to detect calcification below tens of micrometers.^48^, ^49^ As such, if female patients have more significant microscale calcification as our data suggest, this could provide one explanation for the decreased or delayed diagnosis of AVS in female patients that ultimately result in similar severity levels to male patients, but with reduced calcification.^50^

Collectively, we show that females have increased levels of OPN expression, as shown in both human valve tissue (Figure 2) and within our in vitro hydrogel cultures using female porcine cells (Figure 3). Although only OPN mRNA, and not protein, was significantly increased in our hydrogel system, this may be related to OPN's diverse post‐translational modifications and functions. Even after protease digestion, OPN fragments retain biological activity,^51^ meaning that cell‐secreted thrombin and MMPs can dramatically alter the availability of the complete OPN protein while lower molecular weight cleavage products preserve its cell‐binding function even in the absence of its RGD and polyaspartate sequences.^30^ These fragments have been observed to contain newly exposed active domains^52^ and may participate in unexplored feedback loops between MMP‐mediated ECM remodeling, calcium deposition, and OPN secretion, sequestration, and digestion. As such, full‐length OPN protein may be a poor proxy for OPN activity. Regardless, OPN mRNA has been broadly used to examine OPN gene expression and correlates with disease pathogenesis and homeostatic osteogenesis in a variety of contexts.^31^, ^53^, ^54^, ^55^, ^56^, ^57^

Few published results demonstrate sex differences in cellular responses to OPN; however, OPN has been found to inhibit atherosclerosis in female apolipoprotein (apo) E‐deficient mice relative to male.^28^ Evidence also supports the role of female sex hormones in inhibition of OPN expression. A study using bovine coronary smooth muscle cells showed that exogenous treatment with β‐estradiol in the presence of osteogenic medium resulted in decreased OPN mRNA levels, but increased calcification.^58^ Likewise, OPN mRNA was shown to be increased in renal tissue in ovariectomized rats.^59^ Thus, a decrease of estrogens, such as post‐menopause, might result in increased expression of OPN as a compensatory mechanism. The OPN promoter gene contains four single nucleotide polymorphisms that regulate the activity of OPN and may be involved in sexual dimorphisms in hepatocellular carcinoma development in patients with hepatitis C infection.^60^ Additionally, in response to the exogenous delivery of ET‐1, we observed a decrease in calcification of encapsulated male VICs accompanied with an increase in OPN mRNA, and a shift in localization of OPN to perinuclear regions (Figure 6). We suspect ET‐1 is a critical regulator of sex‐specific fibro‐calcification, as our current work demonstrates a link between ET‐1 and OPN expression, and previous work suggests that ET‐1 increases myofibroblast activation in female VICs via genes that escape XCI. The results presented in this manuscript are some of the first studies examining the sex‐specific regulation of OPN in the context of ET‐1, a biochemical factor critical to driving AVS progression.

We also showed that OPN increased, but calcification decreased, in both human tissues and in a PEG + Col hydrogel as a simplified model of AVS (Figures 3 and 4). One explanation for our observations might be related to the increased presence of a rich and disorganized collagen matrix in female valve tissue that was observed in Figure 2. Collagen type I is known to bind OPN,^45^ potentially leading to increased sequestration of full‐length OPN within female valves. Future experiments might focus on the changes in phosphorylation states and other post‐translational modifications between ECM and nuclear localization of OPN, as this could provide insight as to how OPN may be mediating local mineralization. The phosphorylation state of OPN is crucial in determining its biological function, making exogenous delivery for the inhibition of matrix mineralization difficult,^29^, ^30^, ^51^, ^61^ and presenting additional potential therapeutic targets in the form of the secreted kinases that regulate OPN phosphorylation.^62^, ^63^ In vivo, the hypothesis is that OPN reduces calcification of aortic valves by recruiting macrophages to the region of ectopic calcification, increasing the local pH, and dissolving accumulated calcification.^26^, ^64^ With an increased appreciation of sex as a variable in reducing valve calcification in females, as observed in clinical studies, we posit that increased production of OPN by female VICs may recruit more macrophages to the diseased valve tissue, and further studies are warranted on this topic.

The PEG + Col hydrogel system employed here demonstrates sexual dimorphisms in VIC cultures that are relevant to clinical data and confirmed using human diseased tissue. Interestingly, the sex‐specific trends of OPN mRNA expression in response to ET‐1 treatment in 3D hydrogels were not the same for male and female VICs plated on TCPS (Figure S5), suggesting the importance of designing in vitro studies to mimic the dimensionality of native tissue. Considering the physical size and timescale of these 3D cultures and the sensitivity of primary VICs to transfection, RNAi treatment or gene knockout of OPN were beyond the scope of this study. Regardless, the role of ET‐1 as an inducer of OPN and OPN's subsequent role in regulating ECM remodeling, fibrosis, and calcification has been demonstrated in a variety of cell and tissue types, including human myocardium.^31^, ^32^, ^65^ OPN primarily localized within the ECM of female samples in OM but shifted to areas nearby the cell nuclei when exposed to calcific culture conditions, triggering microscale matrix mineralization. The change in localization of OPN in females led us to believe that OPN transitioning from the ECM to regions of calcification may play a role in preventing mineralization growth or reduce the number of nucleation sites, thus leading to the reduced calcification observed in females, both in vitro and in vivo. Likewise, OPN changed in localization from the ECM to perinuclear regions in male samples exogenously treated with ET‐1 that exhibited reduced calcification, further supporting this hypothesis. Moving forward, these and other advanced culture models should prove useful to test detailed hypotheses related to sex‐specific AVS progression and calcification, and guide drug discovery and development for sex‐specific treatments.

As the sample size of human tissues was limited to only two male and three female aortic valves in this study, these results are restricted in their statistical power and are not intended to be conclusive. However, the trends observed in these valve sections regarding sex‐specific fibrotic vs. calcific phenotypes and OPN expression were recapitulated in the 3D hydrogel cultures. Moreover, sex hormones have been shown to regulate both serum ET‐1 levels and calcium content in atherosclerotic lesions in vivo,^66^, ^67^, ^68^ suggesting that age‐related changes in estrogen and testosterone may control disease pathogenesis and progression. An inverse relationship between collagen content and calcification area has been demonstrated in human carotid artery plaques and a corresponding 3D hydrogel model,^49^ underscoring the interplay between ECM composition and disease phenotype and reinforcing our finding that sex‐specific matrix remodeling has distinct effects on calcification, which is in turn attenuated by ET‐1 exposure. Recently, a survey of more than 200 human aortic valves collected over a 7‐year period identified OPN as being increased in male tissues.^69^ This study focused on a European patient population and relied on 2D cultures on TCPS and limited methodologies, which could contribute to differences seen in results from histological and transcriptomic analyses. Taken together, our work and ongoing research in the field collectively highlight the need for continued research to parse the relative contributions of age, genetics, and lifestyle on sex‐specific aortic valve disease phenotypes.

## 5 CONCLUSION

We recapitulate results presented in clinical data in that female patients have smaller scale microcalcifications compared to male patients. Initially, we demonstrated a reduction in overall calcification and in screening for other markers of VIC osteoblast‐like phenotype, we observed an increase in expression of OPN in female valve tissue sections. Using a 3D PEG + Col hydrogel system to encapsulate female and male VICs, we were able to recapitulate findings from human tissue samples in vitro and test specific mechanistic hypotheses. Specifically, we saw an increase in female VIC OPN expression and noted that OPN deposition in female samples shifted from the ECM to areas surrounding the cell nuclei, which was concurrent with small‐scale matrix mineralization. The shift in localization of OPN, accompanied with reduced calcification, was observed in male VIC samples treated exogenously with ET‐1 as an upstream target of OPN expression. These results suggest that OPN may inhibit mineral growth and expansion in a sex‐specific manner and highlights the value of in vitro culture platforms to study clinically relevant sex dimorphisms.

## AUTHOR CONTRIBUTIONS

**Megan E. Schroeder:** Conceptualization (lead); data curation (lead); formal analysis (lead); funding acquisition (supporting); investigation (lead); methodology (lead); validation (lead); visualization (lead); writing – original draft (lead); writing – review and editing (lead). **Dilara Batan:** Formal analysis (supporting); investigation (supporting); writing – review and editing (supporting). **Andrea Gonzalez Rodriguez:** Conceptualization (supporting); data curation (supporting); methodology (supporting). **Kelly F. Speckl:** Data curation (supporting); formal analysis (supporting). **Douglas K. Peters:** Data curation (supporting); formal analysis (supporting); software (lead). **Bruce E. Kirkpatrick:** Formal analysis (supporting); software (supporting); visualization (equal); writing – review and editing (supporting). **Grace K. Hach:** Formal analysis (supporting); writing – review and editing (supporting). **Cierra J. Walker:** Conceptualization (supporting); formal analysis (supporting); writing – review and editing (supporting). **Joseph C. Grim:** Conceptualization (supporting); writing – review and editing (supporting). **Brian A. Aguado:** Conceptualization (supporting); writing – review and editing (supporting). **Robert M. Weiss:** Conceptualization (supporting); funding acquisition (supporting); resources (supporting); writing – review and editing (supporting). **Kristi S. Anseth:** Conceptualization (supporting); resources (lead); supervision (lead); writing – review and editing (equal).

## CONFLICT OF INTEREST

The authors declare no conflict of interest.

### PEER REVIEW

The peer review history for this article is available at https://publons.com/publon/10.1002/btm2.10358.

## Supporting information


**Appendix S1** Supporting Information

## Data Availability

The data that support the findings of this study are available from the corresponding author upon reasonable request.
