# Supplementary material for: Osteopontin activity modulates sex‐specific calcification in engineered valve tissue mimics
Source: Bioeng Transl Med. 2022 Jun 15;8(1):e10358. doi: 10.1002/btm2.10358 (PMC9842038; doi:10.1002/btm2.10358)
Supplement: Supplementary file 1 — Appendix S1 Supporting Information [file BTM2-8-e10358-s001.docx]

**Supplemental Materials**

Osteopontin activity modulates sex-specific calcification in engineered valve tissue mimics

Megan E. Schroeder, Dilara Batan, Andrea Gonzalez Rodriguez, Kelly F. Speckl, Douglas K. Peters, Bruce E. Kirkpatrick, Grace K. Hach, Cierra J. Walker, Joseph C. Grim, Brian A. Aguado, Robert M. Weiss, Kristi S. Anseth

| Sex | Age | Cat. No. | Sample ID | Case ID | Case Notes |
| --- | --- | --- | --- | --- | --- |
| Male | 76 | CS508070 | FR0001995D | CI0000005759 | Heart valve replacement; coronary artery disease; hypertension; benign prostatic hypertrophy; myocardial infarction; diabetes mellitus; coronary artery bypass graft; high cholesterol |
| Male | 57 | CS515588 | FR5B339286 | CI0000007348 | Heart valve repair; hypertension, intermittent  claudication, shortness of breath, Ross procedure with aortic valve insufficiency and root dilatation |
| Female | 65 | CS515622 | FR000243F1 | CI0000007350 | Aortic valve replacement; critical aortic stenosis; glaucoma, heart murmur, obesity, hypercholesterolemia |
| Female | 63 | CS509731 | FR0001CA83 | CI0000006139 | N/A |
| Female | 57 | CS511368 | FR0001E235 | CI0000006732 | Heart valve excision; mitral regurgitation, aortic stenosis |

**Supplemental Table 1.** Patient information for the flash frozen human aortic valve tissue samples used in this study. Tissues purchased from OriGene.

**Supplemental Figure 1.** Autocorrelation analysis pipeline. A) Representative image from patient 995D. B) Image converted to 8-bit image type. C) Thresholded 8-bit image to produce a binary image of VK stain; yellow circle represent radial correlation length. D) Result of radial autocorrelation analysis on binary image; area under curve (yellow) calculates a correlation length of ~55 µm, representing the largest radial feature size in the image that retains high autocorrelation (yellow circle in panel C).

**Supplemental Figure 2. Porcine tissues from healthy pigs show no differences in OPN immunofluorescent staining.** Representative images from porcine aortic valve sections from freshly isolated tissue (24-hours post slaughter) and stained for OPN (red) and nuclei (blue) for female (A) and male (B) tissue section, C) Quantification of the OPN mean fluorescence resulted in a non-significant difference between the female and male porcine aortic valve tissue sections. Scale bar = 100 μm. (n= 3 porcine donors for each sex; 10 images per donor minimum).

**Supplemental Figure 3. Colorimetric calcium assay shows increase in deposited calcium in hydrogels cultured in calcifying medium (CM) relative to osteogenic medium (OM).** Calcium assay for Ca^2+^ content deposited within hydrogels at day 7 and day 12, showing no significant differences between sexes for samples in CM or OM. CM resulted in a significant increase in calcium relative to OM at both timepoints.

**Supplemental Figure 4**. **Representative images demonstrating MatLab image analysis used to determine the localization of the OPN fluorescence intensity**. Here, histological sections from female VICs in the PEG + Col hydrogel treated with either OM (osteogenic medium) or CM (calcifying medium) were stained for OPN (red) and nuclei (DAPI, gray). Briefly, a median intensity z-projection was created, and the DAPI signal was used to segment nuclear ROIs. The nuclear signal was dilated to include a small number of adjacent, "cellular" pixels. Mean OPN signal intensity was calculated for the cellular area (inside the cyan ROIs above) relative to the ECM signal (all the remaining pixels). Data were presented as cell:ECM OPN signal.

**Supplemental Figure 5**. A) OPN gene expression for female and male VICs grown on tissue culture polystyrene (TCPS) in CM (OM (osteogenic medium) + 1 mg/mL CaCl_2_) with indicated concentration of ET-1 for 3 days. Gene expression normalized relative to L30 control. B) OPN fluorescent mean integrated density of PEG + Col samples containing female or male VICs cultured in CM with indicated concentration

**Supplemental Discussion on Spatial Autocorrelation.** As is demonstrated in Figs. 1C, 3G, 4C, and 6C, VK density is a common quantitative assessment for this stain and can be useful for measuring differences in, effectively, signal intensity. However, this measurement does not account for the spatial distribution of this signal, as is illustrated in Figs. 6B and 6C. Comparing between control (0 nM ET-1) conditions in females and males shows no difference in VK density, but the distribution of this signal is distinct between sexes. Moreover, as is seen in Fig. 1C, VK density does not identify significant differences between highly aggregated and more sparsely distributed signal when these regions of positivity have comparable intensity. While attempts can be made to quantify the relative area distributions of these regions, interpreting this data requires binning or other user-enforced manipulations. For example, using MATLAB to bin the areas of positive VK stain in healthy vs. sex-specific AVD produced the following plot:

While this analysis demonstrates some qualitative differences (males have more “large” aggregates >3200 µm^2^ and fewer “small” aggregates <800 µm^2^), these differences are partly dictated by bin widths, which are ultimately arbitrary and lack physiological relevance. After consulting a wide variety of data processing approaches, we took inspiration from studies using spatial autocorrelation to measure homogeneity and porosity for their relevance to the mineralization processes evoked by calcific disease.^43,44^ This strategy overcomes two important limitations of the previously described techniques (VK density and area distribution). First, spatial autocorrelation considers the geometric distribution of positive signal, which is lost with traditional VK analysis. Spatial autocorrelation can identify differences in shape, size, and arrangement as all these features contribute to the positional comparisons between regions of positive signal that comprise this measurement. Second, spatial autocorrelation analysis and subsequent integration to tabulate the correlation length calculates a physically relevant, single-term parameter that requires no additional user manipulation, in contrast to area distribution measurements that often rely on the arbitrary threshold values mentioned above. Instead, the correlation length is a quantitative measure of spatial segregation between positive features with broad utility ranging from determining the length scale of ordered structures in multicellular constructs^1-5^ to identifying geometric differences in tissue biopsies to diagnose and differentiate cancerous lesions.^6-8^

**References**

1. Guillamat P, Blanch-Mercader C, Pernollet G, Kruse K, Roux A. Integer topological defects organize stresses driving tissue morphogenesis. *Nat Mater*. Feb 10 2022;doi:10.1038/s41563-022-01194-5

2. Saraswathibhatla A, Zhang J, Notbohm J. Coordination of contractile tension and cell area changes in an epithelial cell monolayer. *Phys Rev E*. Feb 2022;105(2-1):024404. doi:10.1103/PhysRevE.105.024404

3. Vazquez K, Saraswathibhatla A, Notbohm J. Effect of substrate stiffness on friction in collective cell migration. *Sci Rep*. Feb 15 2022;12(1):2474. doi:10.1038/s41598-022-06504-0

4. Mongera A, Rowghanian P, Gustafson HJ, et al. A fluid-to-solid jamming transition underlies vertebrate body axis elongation. *Nature*. Sep 2018;561(7723):401-405. doi:10.1038/s41586-018-0479-2

5. Lenne PF, Trivedi V. Sculpting tissues by phase transitions. *Nat Commun*. Feb 3 2022;13(1):664. doi:10.1038/s41467-022-28151-9

6. Robles FE, Deb S, Wilson JW, et al. Pump-probe imaging of pigmented cutaneous melanoma primary lesions gives insight into metastatic potential. *Biomed Opt Express*. Sep 1 2015;6(9):3631-45. doi:10.1364/BOE.6.003631

7. Robles FE, Wilson JW, Warren WS. Quantifying melanin spatial distribution using pump-probe microscopy and a 2-d morphological autocorrelation transformation for melanoma diagnosis. *J Biomed Opt*. Dec 2013;18(12):120502. doi:10.1117/1.JBO.18.12.120502

8. Zaffar M, Pradhan A. Spatial autocorrelation analysis on two-dimensional images of mueller matrix for diagnosis and differentiation of cervical precancer. *J Biophotonics*. Jul 2020;13(7):e202000006. doi:10.1002/jbio.202000006
